# Harnessing AI for advancing pathogenic microbiology: a bibliometric and topic modeling approach

**DOI:** 10.3389/fmicb.2024.1510139

**Published:** 2024-11-15

**Authors:** Tian Tian, Xuan Zhang, Fei Zhang, Xinghe Huang, Minglin Li, Ziwei Quan, Wenyue Wang, Jiawei Lei, Yuting Wang, Ying Liu, Jia-He Wang

**Affiliations:** ^1^Department of Family Medicine, Shengjing Hospital of China Medical University, Shenyang, China; ^2^College of Metrology and Measurement Engineering, China Jiliang University, Hangzhou, China; ^3^Department of General Practice, The First Hospital of China Medical University, Shenyang, China; ^4^Department of Cardiology, Shengjing Hospital of China Medical University, Shenyang, Liaoning, China; ^5^Department of Nephrology, Shengjing Hospital of China Medical University, Shenyang, China

**Keywords:** pathogenic microorganisms, artificial intelligence (AI), machine learning (ML), deep learning (DL), bibliometrics, topic modeling, antimicrobial resistance (AMR)

## Abstract

**Introduction:**

The integration of artificial intelligence (AI) in pathogenic microbiology has accelerated research and innovation. This study aims to explore the evolution and trends of AI applications in this domain, providing insights into how AI is transforming research and practice in pathogenic microbiology.

**Methods:**

We employed bibliometric analysis and topic modeling to examine 27,420 publications from the Web of Science Core Collection, covering the period from 2010 to 2024. These methods enabled us to identify key trends, research areas, and the geographical distribution of research efforts.

**Results:**

Since 2016, there has been an exponential increase in AI-related publications, with significant contributions from China and the USA. Our analysis identified eight major AI application areas: pathogen detection, antibiotic resistance prediction, transmission modeling, genomic analysis, therapeutic optimization, ecological profiling, vaccine development, and data management systems. Notably, we found significant lexical overlaps between these areas, especially between drug resistance and vaccine development, suggesting an interconnected research landscape.

**Discussion:**

AI is increasingly moving from laboratory research to clinical applications, enhancing hospital operations and public health strategies. It plays a vital role in optimizing pathogen detection, improving diagnostic speed, treatment efficacy, and disease control, particularly through advancements in rapid antibiotic susceptibility testing and COVID-19 vaccine development. This study highlights the current status, progress, and challenges of AI in pathogenic microbiology, guiding future research directions, resource allocation, and policy-making.

## 1 Introduction

Pathogenic microorganisms, including viruses, bacteria, fungi, and parasites, cause infections and diseases in hosts. Since the 1960s, the widespread use of antibiotics has driven the evolution of these microorganisms through natural selection, gene recombination, and horizontal gene transfer (HGT), leading to antibiotic resistance (AMR).

AMR results in millions of deaths annually worldwide, posing a severe threat to public health (Saha and Sarkar, 2021; Uddin et al., 2021). Traditional culture-based methods fail to address the increasing genetic diversity and resistance of pathogens. In the era of big data, research on pathogenic microorganisms heavily relies on high-throughput sequencing, metagenomics, proteomics, and targeted techniques (Lewis et al., 2021; Wani et al., 2022). Effectively organizing, analyzing, and interpreting the vast amounts of biomedical data generated has emerged as a new challenge.

AI, a field that simulates and extends human intelligence through computational devices, provides powerful tools to address these challenges. Machine learning (ML) improves computer performance through pattern recognition and analysis, enabling precise microbial classification, biomarker identification, small molecule compound library screening, and novel anti-infective drug discovery.

Deep learning (DL), comprising multilayer neural networks, boosts data generation capabilities for pathogenic microorganisms through neural networks, generative models, and variational autoencoders (Huo and Wang, 2024; Wong et al., 2023). Computer vision (CV) rapidly detects pathogens in microscope or fluorescence sensor images (Zhao et al., 2024; Matias et al., 2021). Natural language processing (NLP) automatically identifies information from scientific literature on pathogen research (Jimeno-Yepes and Verspoor, 2023) and analyzes bacteriophage genomes to predict their life cycles (Tynecki et al., 2020).

The application of AI in pathogenic microbiology has been widely explored, with many scholars evaluating its use in related research. Literature reviews date back to 2014. Specifically, Nourani et al. studied ML, homology prediction, and structural prediction in predicting pathogen-host protein interactions (PHI) between 2009 and 2014, crucial for understanding infection mechanisms (Nourani et al., 2015). Rondon-Villarreal et al. reviewed ML in antimicrobial peptide design, a potential new class of antimicrobial drugs to combat AMR (Rondon-Villarreal et al., 2014). Qu et al. comprehensively reviewed ML in microbiology, covering microbial classification from high-throughput sequencing data, environmental and host phenotype prediction, and microbial-disease association analysis (Qu et al., 2019). Agany et al. explored data mining and ML in understanding vector-host-pathogen relationships from 2012 to 2020, highlighting advances in DL and association rule analysis (Agany et al., 2020). Peiffer-Smadja et al. studied ML in clinical microbiology, identifying 97 ML systems aimed at assisting clinical microbiologists with bacterial, parasitic, viral, and fungal infection analysis and antimicrobial sensitivity assessment up to 2020 (Peiffer-Smadja et al., 2020). Pillai et al. summarized various AI models (e.g., logistic regression, random forests, support vector machines, neural networks, ensemble methods) in predicting zoonotic disease outbreaks and identifying risk factors (Pillai et al., 2022). He et al. introduced AI’s role in infectious disease drug delivery, including drug development, resistance prediction, dose optimization, and drug combination selection (He et al., 2021). Hu et al. discussed ML’s broad applications in protozoan pathogen and infectious disease research, covering detection, diagnosis, monitoring, host–parasite interactions, drug discovery, and vaccine development (Hu et al., 2022). Kaur et al. reviewed AI techniques in predicting and monitoring vector-borne diseases and their pathogens, noting significant progress in disease prediction, vector identification, and outbreak monitoring through ML and DL (Kaur et al., 2022).

Despite several studies exploring the application of AI in specific areas of pathogenic microbiology, a systematic analysis of the overall development trends and knowledge structure of the field is lacking. Previous literature reviews have primarily focused on AI’s performance in specific application scenarios. These studies provide important references for understanding AI’s value in specific applications but fail to offer a comprehensive grasp of the overall development landscape of AI in pathogenic microbiology research.

In this context, bibliometrics and topic modeling offer powerful methods to explore and understand scientific research in this domain. Bibliometrics, a statistical method widely used to analyze publication trends and relationships in the medical field, includes evaluative and relational bibliometrics. The latter reveals hidden relationships and research status by analyzing metadata from authors, papers, and journals (Ninkov et al., 2022). Topic modeling, a natural language processing technique, identifies latent semantic patterns in document collections, helping researchers discover cross-disciplinary themes and research trends. Latent Dirichlet Allocation (LDA) is the most widely utilized technique for this purpose (Vayansky and Kumar, 2020). This study aims to conduct a large-scale quantitative analysis of AI applications in the field of pathogenic microbiology through bibliometrics and topic modeling methods. Compared to existing studies, this paper has the following innovations and contributions: (1) By utilizing 27,420 publications spanning 2010 to 2024, the study conducts a comprehensive quantitative analysis of the field for the first time, covering a wide scope; (2) The integration of bibliometrics and topic modeling techniques not only reveals research hotspots and trends but also deeply explores the potential knowledge structure; and (3) A systematic review of AI’s advancements in eight major application areas within pathogenic microbiology provides a scientific basis for future research directions and resource allocation.

## 2 Methods

### 2.1 Data collection

To ensure the scientific rigor and authority of the literature review, we retrieved data from the Web of Science Core Collection (WoSCC), the oldest and most widely used research publication and citation database globally (Birkle et al., 2020). The citation index includes various versions of WoSCC, such as the Science Citation Index Expanded (SCI-EXPANDED), Social Sciences Citation Index (SSCI), Current Chemical Reactions (CCR-EXPANDED), and Index Chemicus (IC).

The search terms were derived from key phrases mentioned in previous review articles on AI applications in pathogenic microbiology (Table 1). The final search string was: TS = (“Pathogen-host protein–protein interactions” OR Host OR Pathogen OR “Drug Resistance” OR “Antimicrobial Peptides” OR Viruses OR Bacteria OR Fungi OR “Vector-Host-Pathogen Relationships” OR Vector OR Parasites OR “Infectious Diseases” OR “Pathogenic Microbes”).

**Table 1:** Sources of search terms for this study.

Author	Paper title	Keywords related to AI	Keywords related to pathogenic microorganisms	Year
Nourani	Computational approaches for prediction of pathogen-host protein–protein interactions	Homology-based prediction Structure-based prediction	Pathogen-host protein–protein interactions Host Pathogen	2015
Rondon-Villarrea	Machine Learning in the Rational Design of Antimicrobial Peptides	Machine learning	Drug Resistance Antimicrobial Peptides	2014
Qu	Application of Machine Learning in Microbiology	Supervised Learning Unsupervised Learning Support Vector Machine Naïve Bayes Random Forest K Nearest Neighbor	Viruses Bacteria Fungi	2019
Agany	Assessment of vector-host-pathogen relationships using data mining and machine learning	Data Mining Deep Learning Association Rule Mining	Vector-Host-Pathogen Relationships Vector	2020
Peiffer-Smadja	Machine learning in the clinical microbiology laboratory: has the time come for routine practice?	Artificial Neural Network Support Vector Machine Logistic Regression K-nearest Neighbors Decision\Regression\Classification Trees Gradient Boosting Adaptive Boosting	Parasites	2020
Pillai	Artificial Intelligence Models for Zoonotic Pathogens: A Survey	eXtreme Gradient Boosting Long Short Term Memory network Generative Adversarial Network Auto-Encoder		2020
He	Artificial intelligence and machine learning assisted drug delivery for effective treatment of infectious diseases		Infectious Diseases	2021
Hu	Machine Learning and Its Applications for Protozoal Pathogens and Protozoal Infectious Diseases	Convolutional Neural Networks		2022
Kaur	Artificial Intelligence Techniques for Predictive Modeling of Vector-Borne Diseases and its Pathogens: A Systematic Review	Ensemble Classifiers Support Vector Machine	Pathogenic Microbes	2022

AND TS = (“Deep Learning” OR “Association Rule Mining” OR “Artificial Neural Network” OR “Support Vector Machine” OR “K-nearest Neighbors” OR “Decision Trees” OR “Regression Trees” OR “Classification Trees” OR “Gradient Boosting” OR “Adaptive Boosting” OR “eXtreme Gradient Boosting” OR “Long Short Term Memory network” OR “Generative Adversarial Network” OR “Auto-Encoder” OR “Convolutional Neural Networks” OR “Ensemble Classifiers” OR “Support Vector Machine”). This search yielded 151,593 results.

#### 2.1.1 Screening criteria

Inclusion and exclusion criteria were established to filter the results. The inclusion criteria focused on research articles related to AI and pathogenic microorganisms. Exclusion criteria were set for outdated articles, non-English literature, irrelevant disciplines, conference abstracts, and duplicate documents. The screening was performed independently by two authors to ensure accuracy and consistency. The specific steps were:

1. **Year Limitation:** Restricting the publication years to 2010–2024 eliminated 4,858 articles. This timeframe was selected because the rapid advancements in AI and pathogenic microbiology, particularly the widespread application of metagenomics and high-throughput sequencing, began around 2010 (Sheetal Ambardar et al., 2016; Park et al., 2016).
2. **Language Restriction:** Non-English articles (163) were removed to ensure the inclusion of high-impact research and important results published in major journals.
3. **Disciplinary Focus:** Articles from non-medical fields (78,574), such as engineering or computer science, were excluded. These fields often focus more on technical developments and algorithm optimization, which could introduce noise into the bibliometric analysis.
4. **Document Type:** Only “Article” and “Review Article” categories were included, eliminating 40,457 documents from other types like proceeding papers, book chapters, letters, and news items.
5. **Duplicate Removal:** Using Endnote software, 121 duplicate documents were automatically removed to ensure data uniqueness and completeness.

**Figure 1:**
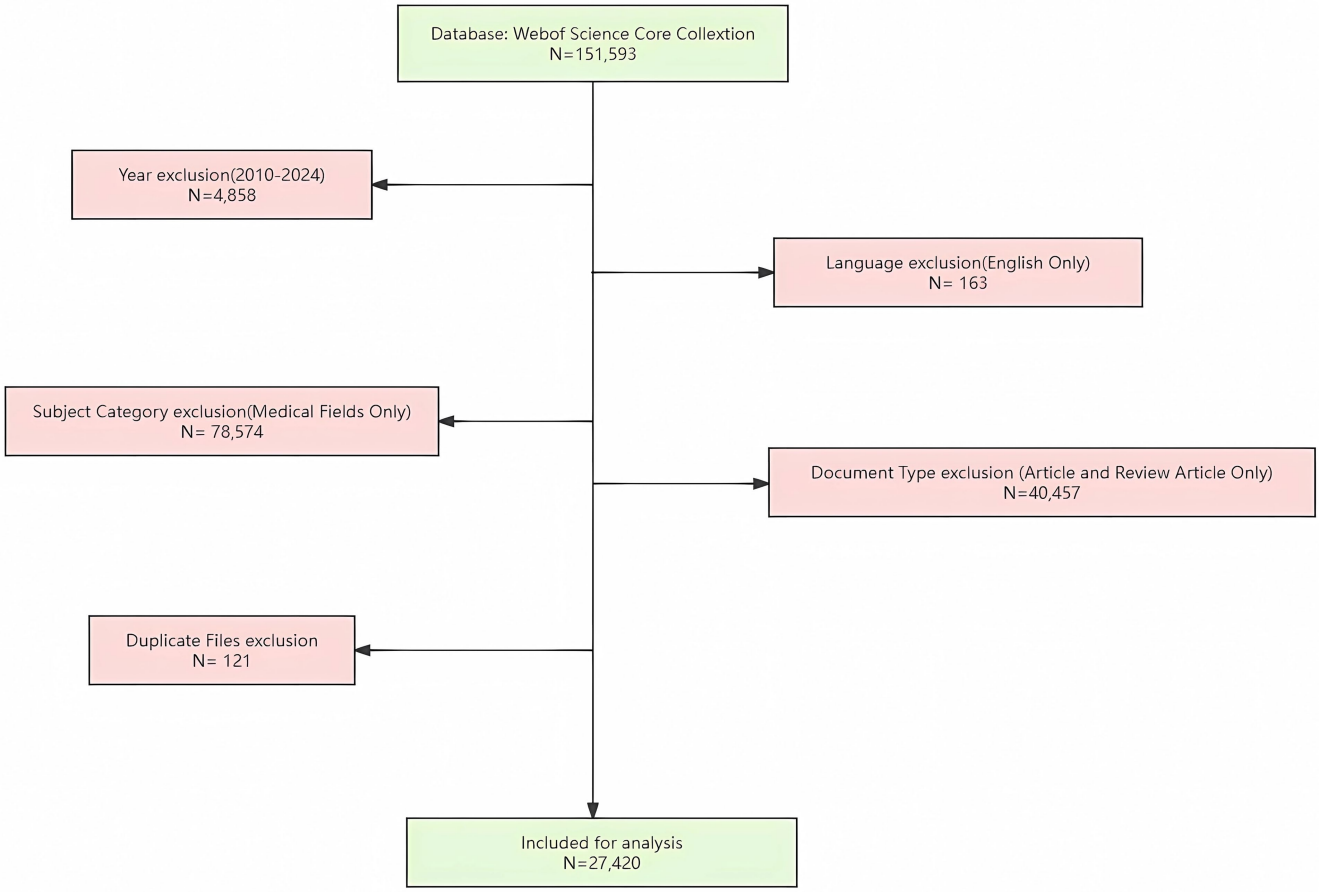
Flow chart of literature search and selection.

The final dataset comprised 27,420 articles for analysis (Figure 1).

### 2.2 Bibliometric analysis

The data was exported in plain text format, including full records and cited references. The clean dataset was imported into three software programs for visualization analysis: R-Bibliometrix 4.3.1, CiteSpace 6.1.R6 (64-bit) Advanced, and VOSviewer 1.6.19.

#### 2.2.1 Bibliometrix

This R-based bibliometric analysis package, launched in 2017, offers robust data processing and multi-dimensional chart generation capabilities (Aria and Cuccurullo, 2017; Arruda et al., 2022). We used Bibliometrix 4.3.1 to analyze annual publications, author H-indices, productivity over time, high-impact journals, and highly cited papers.

#### 2.2.2 CiteSpace

Developed by Professor Chaomei Chen in 2004, CiteSpace is a Java-based scientific literature analysis software capable of document co-citation, collaboration network, and burst term analysis (Chen, 2018). We used CiteSpace 6.1.R6 (64-bit) Advanced to generate co-occurrence maps of institutions.

#### 2.2.3 VOSviewer

Launched in 2010, VOSviewer is a software tool for creating and exploring maps based on network data, offering network, overlay, and density visualizations (Arruda et al., 2022). We used VOSviewer for author and country co-occurrence collaboration analysis.

### 2.3 Topic modeling analysis

Compared to traditional bibliometric keyword clustering methods, topic modeling offers more precise and detailed research classifications, uncovering the underlying structures and dynamic trends within research fields. In our study, we employed Latent Dirichlet Allocation (LDA) for topic modeling using the Python Gensim library. LDA is a generative model that leverages unsupervised machine learning to analyze large volumes of unstructured data, eliminating the need to divide data into training and test sets. It assumes that documents comprise multiple topics, each represented by a probability distribution over words (Chauhan and Shah, 2021; Yang et al., 2024).

Initially, we conducted text preprocessing, including the removal of stopwords and punctuation, as well as stemming, to ensure data consistency and cleanliness. Subsequently, we set the parameters for the topic modeling. Optimization of topics was performed through perplexity and coherence evaluation. Finally, we generated the topic-word distribution and a topic-term relationship network graph.

The specific hyperparameter choices for the LDA model were as follows: alpha = “symmetric” (symmetric prior), and eta = None (default prior). These parameters control the prior beliefs regarding the document-topic and topic-word distributions. The chunksize was set to 2000, meaning that the corpus of 27,420 documents was divided into approximately 14 chunks for processing, thereby avoiding the necessity of loading all documents into memory simultaneously. The passes parameter was set to 1 since the model’s performance was satisfactory with a single pass through the corpus. The model was trained using the LdaModel class provided by Gensim, which implements an online LDA algorithm that enables streaming and incremental training of the corpus, thereby effectively handling large-scale text data. The save and load methods were used for model persistence, ensuring the reproducibility of the experimental results.

## 3 Results

### 3.1 Publication trends analysis

**Figure 2:**
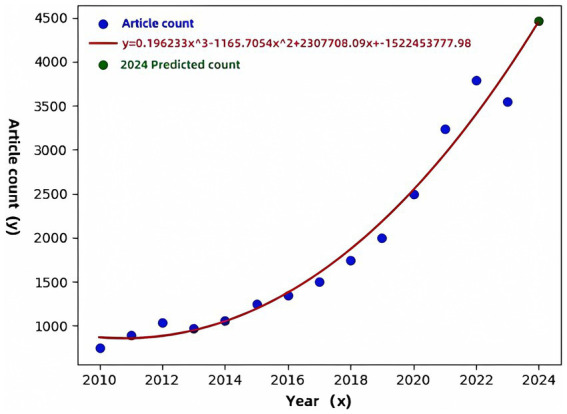
Article quantity trend analysis.

Polynomial regression analysis (Figure 2) from 2010 to 2023 reveals a significant upward trend in the number of publications, with exponential growth evident since 2016. The number of publications is projected to reach approximately 4,500 by 2024. This growth is primarily attributed to the increase in interdisciplinary collaboration, the impact of global health challenges such as the COVID-19 pandemic, and the rapid advancements in AI and computational technologies, particularly breakthroughs in deep learning algorithms, convolutional neural networks, and disease prediction models since 2016 (Jelodar et al., 2019).

### 3.2 Authors

**Figure 3:**
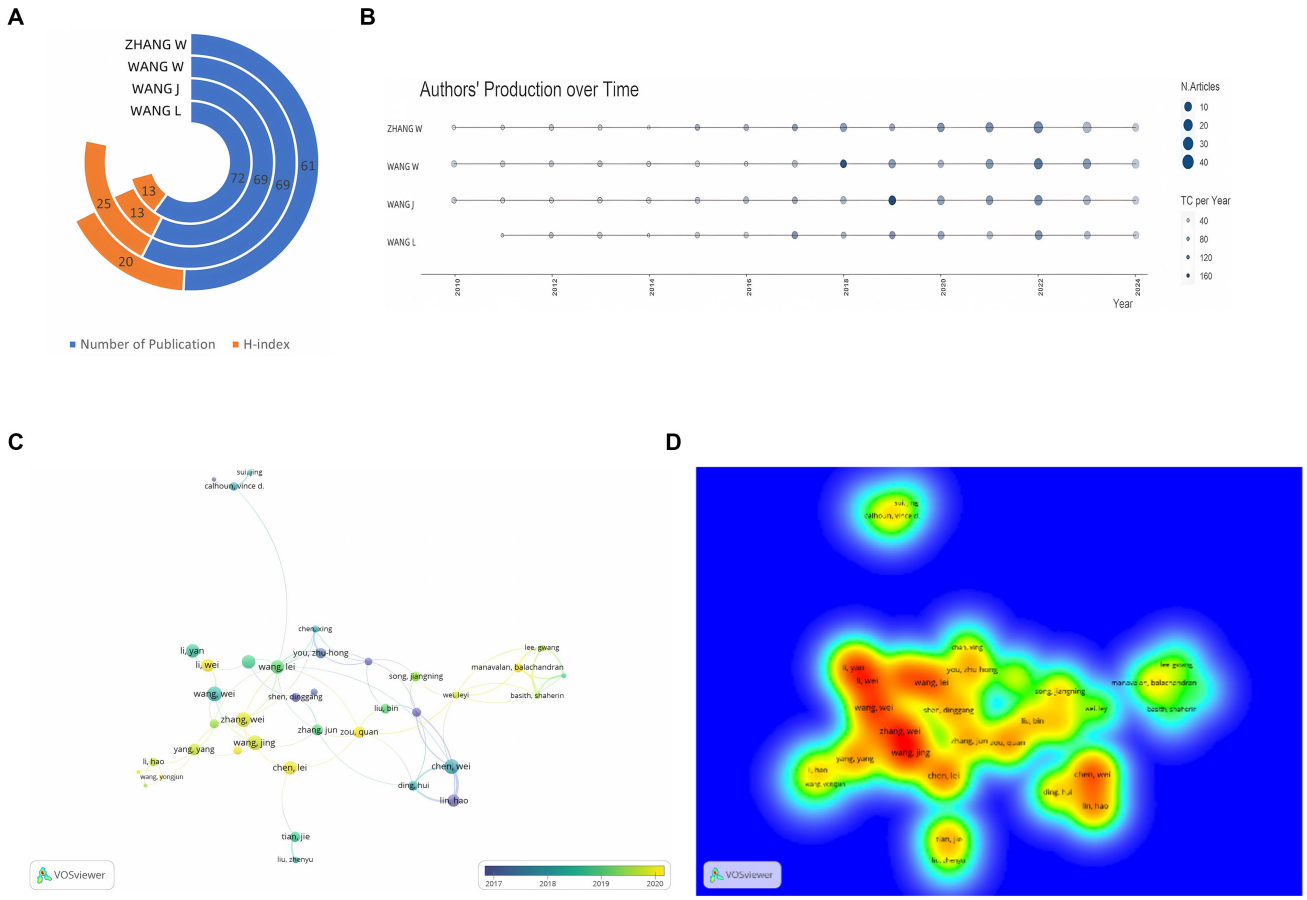
**(A)** Total number of publications and H-index of top 4 authors; **(B)** Authors’ production over time; **(C)** Network visualization of author co-authorship analysis; **(D)** Density visualization of author co-authorship analysis.

Figure 3A presents the metrics for the top four contributing authors. Wang Wei leads with 69 publications and an H-index of 25, signifying that at least 25 publications have been cited at least 25 times (Zhang et al., 2022). Wang Jing was notably prolific in 2018, publishing 11 papers and achieving a Total Citations per Year (TCpY) score of 98.67 (Figure 3B). VOSviewer analysis (Figures 3C,D) of 70 authors, each with a minimum of 5 publications and 1,000 citations, reveals three primary collaborative groups centered around Li Hao, Zhang Wei, and Wang Lie. Notably, Zhang Wei’s collaborative network is the largest, comprising 13 members. The collaboration between Li Hao and Chen Wei is the most frequent, with 28 co-authored papers (Bihari et al., 2023).

### 3.3 Institutions

**Figure 4:**
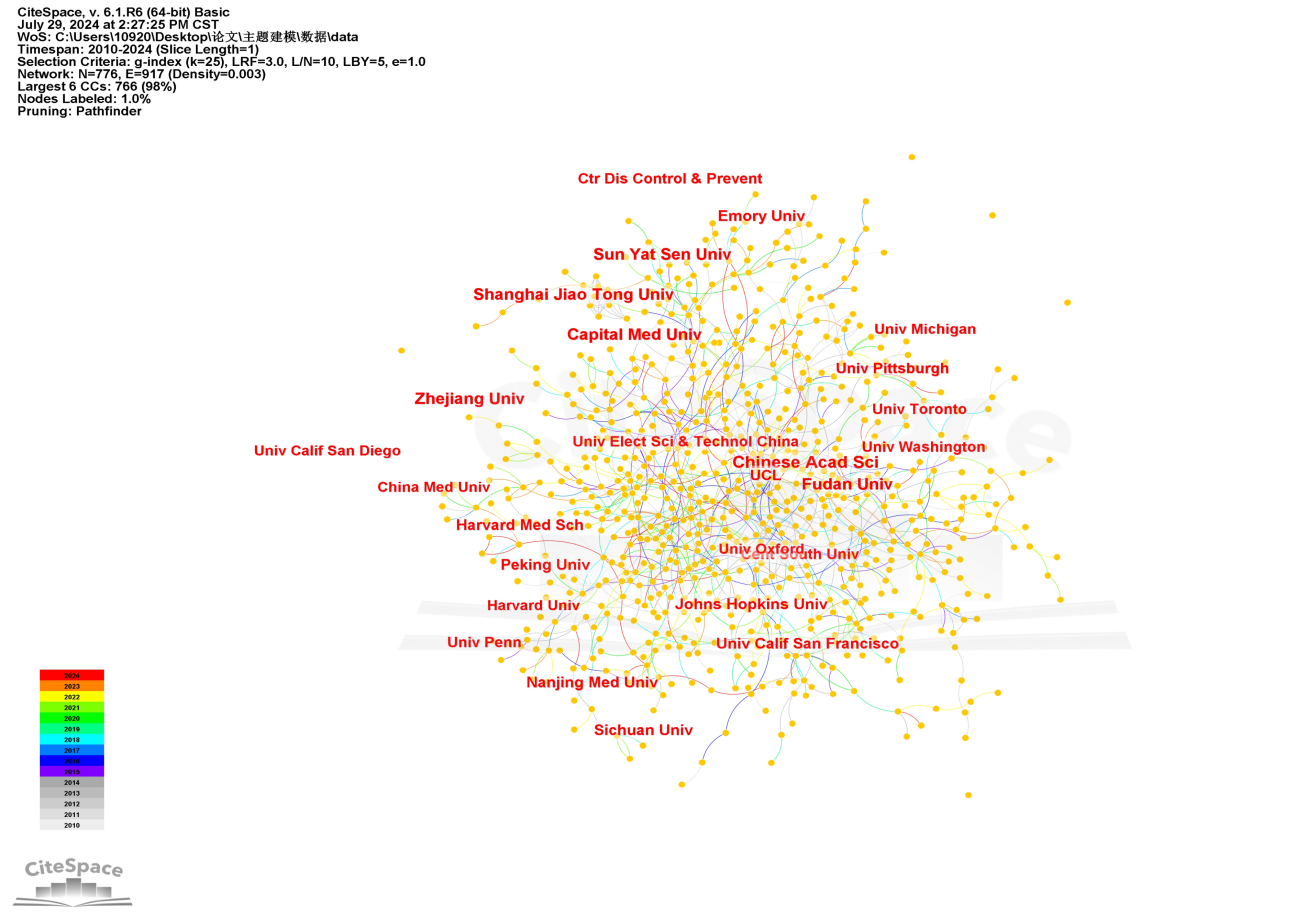
Institution co-occurrence map (node labels: by centrality).

Figure 4 illustrates that there are 917 collaborative interactions among 776 institutions. Although the overall network density is low (0.003), certain institutions display frequent and intensive collaborations. This phenomenon can be attributed to the high specialization in pathogenic microbiology and AI technologies, which leads collaborations to be concentrated among a select few capable institutions. As shown in Table 2, the Chinese Academy of Sciences leads with 486 publications, while the Universitair Medisch Centrum Utrecht demonstrates significant research impact with a betweenness centrality of 0.49.

**Table 2:** The top five institutions by number of publications and intermediate centrality.

Number of publications	Institution
486	Chinese Acad Sci
397	Fudan Univ
383	Capital Med Univ
371	Zhejiang Univ
369	Shanghai Jiao Tong Univ

BC (betweenness centrality)	Institution
0.49	Univ Med Ctr Utrecht
0.35	German Canc Res Ctr
0.28	Second Mil Med Univ
0.28	Univ Amsterdam
0.26	Chinese Acad Sci

### 3.4 Countries

**Figure 5:**
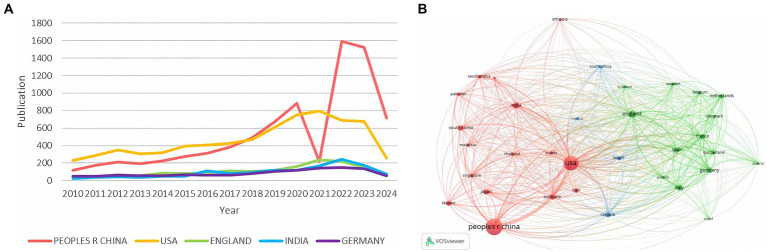
**(A)** Top 5 countries’ production over time; **(B)** Country network visualization.

Figure 5A indicates that China and the United States have been leading in pathogenic microbiology research. Notably, China’s publication volume significantly decreased in 2021, likely due to the impact of the COVID-19 pandemic. However, since 2022, China’s publication rate has grown exponentially, surpassing other countries. VOSviewer analysis (filtering for countries with at least *100 publications) revealed an international collaboration network comprising 32 countries. The thickness of the connecting lines indicates collaboration strength, with China and the U.S. exhibiting the tightest cooperation (link strength = 974). This suggests 974 instances of collaboration between researchers from these two countries, reflecting their central role and significant advantages in knowledge and resource sharing, which are crucial for advancing pathogenic microbiology research and addressing global health challenges (Figure 5B).

### 3.5 Journals

In analyzing the evolution and trends of AI in pathogenic microbiology research, we identified the top ten journals in this field, including their H-index, impact factor, and JCR indicators (Table 3). These metrics reflect the research activity and the journals’ influence within the academic community. “Computers in Biology and Medicine” has the highest number of publications in this field, while “Clinical Infectious Diseases,” the only Q1 journal among the top ten by publication volume, is the most cited, demonstrating its authority. Notably, four of the top ten journals are Q2, indicating that research outcomes are increasingly being published in higher-quality journals.

**Table 3:** Top 10 journals.

Source	Document	Citation	IF	JCR
Computers in Biology and Medicine	455	14,681	7	Q2
BMC Bioinformatics	371	10,308	2.9	Q3
Clinical Infectious Diseases	333	16,107	8.2	Q1
Computer Methods and Programs in Biomedicine	318	10,240	4.9	Q2
Diagnostics	318	2,447	3	Q3
IEEE Computational Intelligence Magazine	302	2,815	10.3	Q2
Frontiers in Oncology	272	2,546	3.5	Q3
IEEE Journal of Biomedical and Health Informatics	228	8,545	6.7	Q2
Bioinformatics	223	10,347	4.4	Q4
Medicine	212	2079	1.3	Q4

### 3.6 Topic modeling

**Figure 6:**
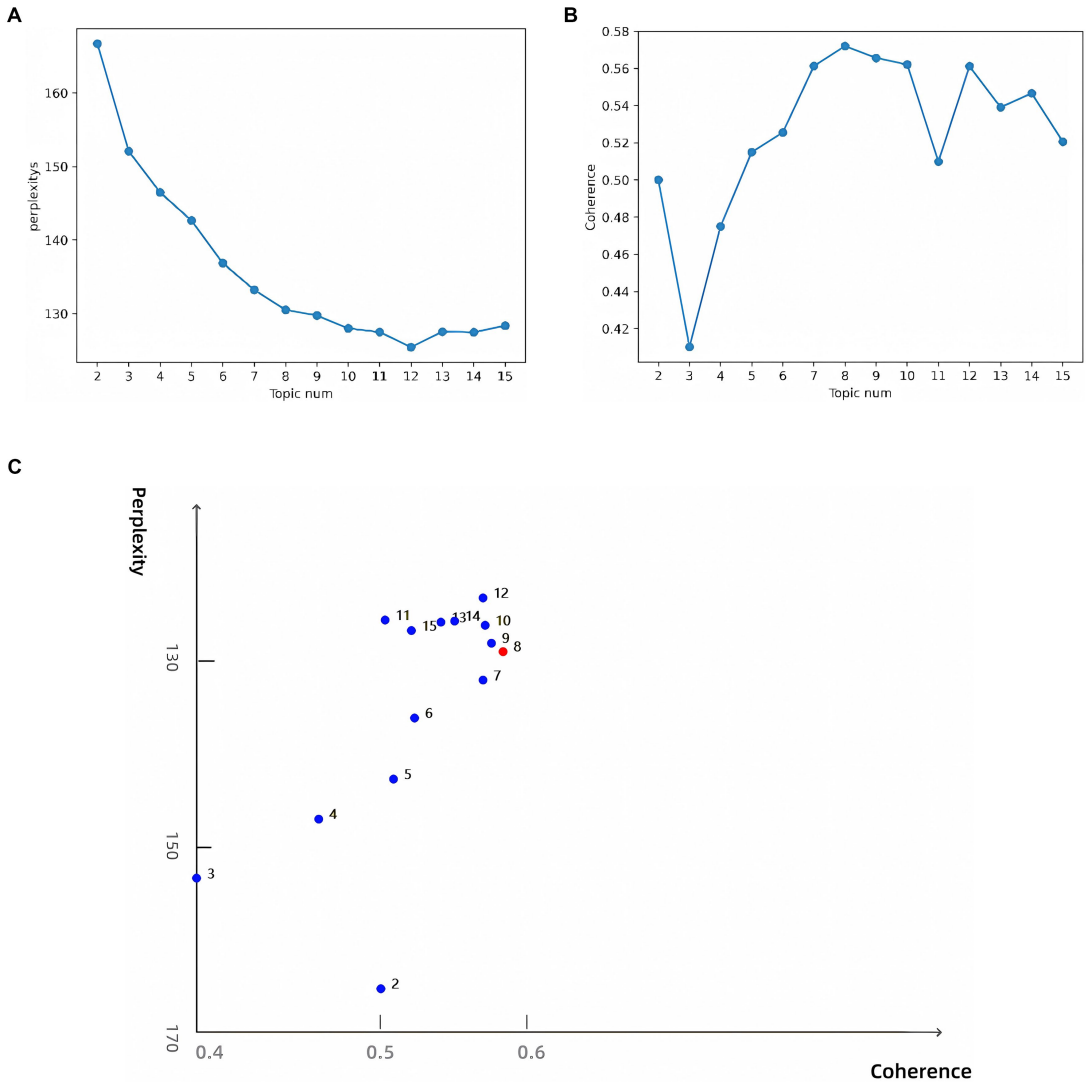
**(A)** Perplexity for topics 2–15; **(B)** Coherence for topics 2–15; **(C)** Topic model optimal parameter selection diagram.

**Figure 7:**
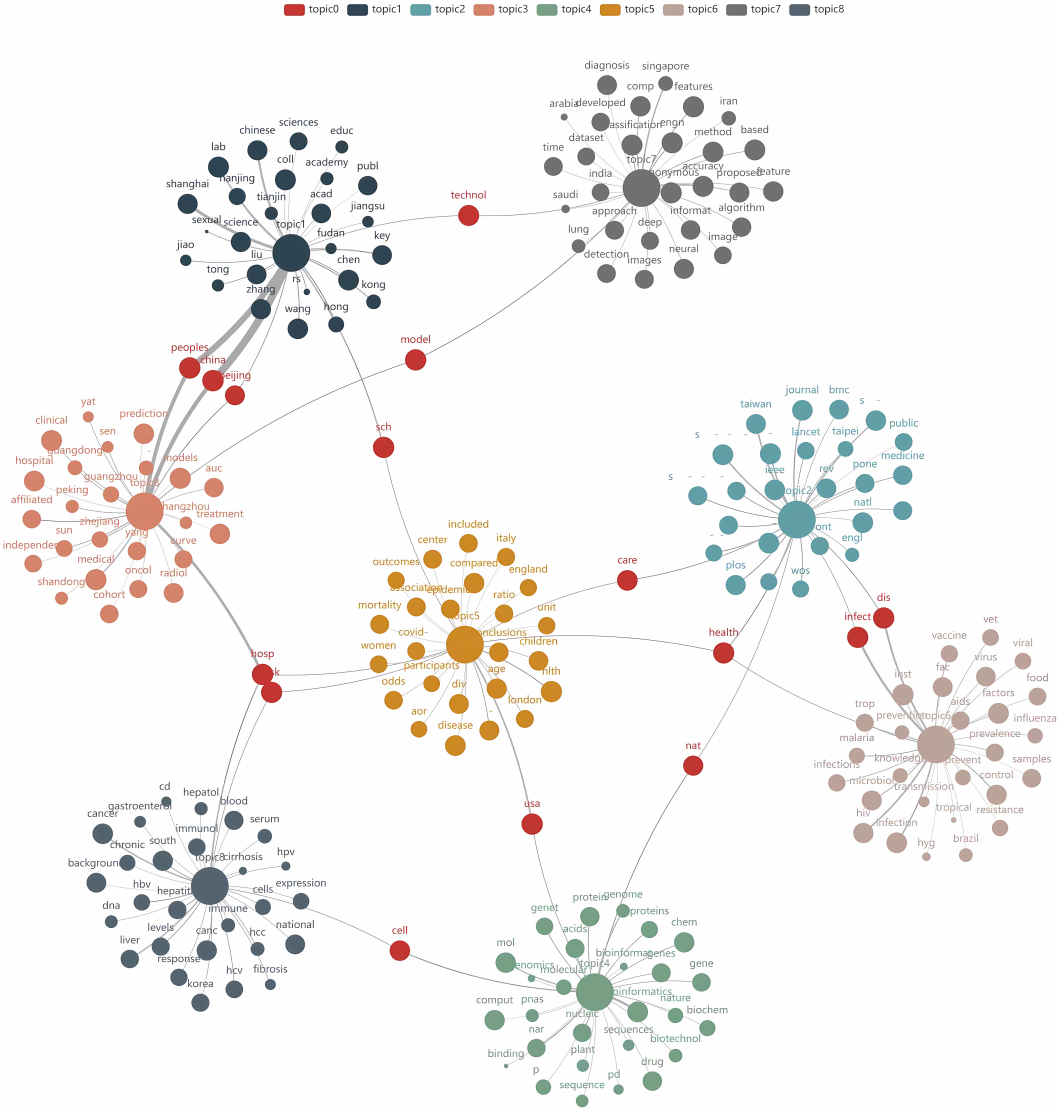
Topic-word relationship diagram.

To determine the optimal number of topics, we undertook the following steps: First, we trained LDA models with varying numbers of topics (2 to 15) and calculated their perplexity scores on the test set. Lower perplexity indicates a better model fit (Figure 6A). Second, we computed the topic coherence score for each model, which measures the semantic consistency of words within a topic; higher values indicate more coherent topic structures (Figure 6B). Finally, we plotted perplexity and coherence scores on a scatter plot (Figure 6C). The top-right region of the plot shows data points for 8, 9, 10, and 12 topics, which performed well in balancing perplexity and coherence. Further manual analysis revealed that although 9, 10, and 12 topics offered higher model performance, they led to overly fine and dispersed classifications, which are impractical for real-world applications. An 8-topic model provided an efficient and practical classification structure, laying a solid foundation for further interpretation. Consequently, we selected the 8-topic model. The resulting themes include AI in pathogen detection, drug resistance, transmission and control, genomics, treatment optimization, ecology, vaccine development, and data analysis and management (Table 4). A word cloud was then utilized to visually represent the intrinsic connections between different research themes, with each cluster marked in different colors. The clusters were interconnected through shared keywords (red nodes), and the size of each node reflected keyword frequency, while the thickness of connecting lines indicated the distribution strength of words within specific topics (Figure 7).

**Table 4:** Topic-word distribution (manually screened).

Theme	Intensity	Distribution
Topic0	0.043646	Recognition, detection, microorganisms, diagnosis, sensitivity, specificity, algorithms, models, deep learning, rapid, automation, pathogens
Topic1	0.225899	Resistance, drugs, genes, sequences, mutations, evolution, prediction, analysis, antibiotic resistance, bacteria, experimental data, antibiotics
Topic2	0.082465	Transmission, infection, control, epidemic, prediction, monitoring, public health, transmission routes, risk assessment, early warning systems, spread
Topic3	0.089147	Genome, sequencing, genes, analysis, microorganisms, diversity, evolution, comparative, database, functional prediction, gene expression
Topic4	0.174416	Treatment, optimization, plans, personalized, efficacy, prediction, assessment, therapeutic outcomes, decision support, patient data, medical protocols
Topic5	0.107509	Ecology, microorganisms, environment, interactions, communities, ecosystems, analysis, monitoring, modeling, biodiversity
Topic6	0.195139	Vaccine, development, antigens, immune response, prediction, experimental data, simulation, efficacy, protection rate, bioinformatics, clinical trials
Topic7	0.08178	Data, analysis, management, databases, informatics, storage, big data, data mining, computation, statistics, automated processing

## 4 Discussion

### 4.1 Eight major topics

This section summarizes the eight topics identified through topic modeling and discusses the research advancements within each domain.

#### 4.1.1 Application of artificial intelligence in pathogen detection

Traditional diagnostic methods, such as microbial culture and isolation, are often time-consuming and prone to false-negative results (Daim et al., 2006). The application of multi-modal data fusion techniques in pathogen detection has gained significant attention in recent years. These techniques integrate image data with genetic data to provide more comprehensive and accurate diagnostic outcomes. For instance, Khan et al. (2019) developed models using automated image capture technology and convolutional neural networks (CNN), successfully classifying and identifying Gram-stained blood cultures. Their model achieved a classification accuracy of 94.9% for both Gram-positive cocci and Gram-negative bacteria. By integrating microbial genome sequencing data with the capabilities of CNNs, researchers can further subtype pathogens based on pattern recognition. This multi-modal fusion approach significantly enhances sensitivity and specificity in diagnostics by simultaneously analyzing the visual and genetic characteristics of pathogens, thus making pathogen diagnosis more efficient and precise.

Additionally, machine learning models can rapidly analyze complex data patterns, thereby improving diagnostic speed and accuracy (Smith et al., 2018). For instance, models used for DNA sequencing can quickly process the genomes of bacteria and viruses (Ali et al., 2023).

#### 4.1.2 Application of artificial intelligence in antimicrobial resistance research

Artificial intelligence has been effectively utilized in the analysis and prediction of microbial drug resistance, marking a significant advancement in antimicrobial resistance research. The increasing prevalence of resistant bacteria underscores the critical importance of analyzing genomic and sequence data. Traditional antibiotic susceptibility testing (AST) methodologies require a minimum of 4 days, which is excessively time-consuming for urgent clinical scenarios where swift decision-making is crucial, especially in the face of rapidly spreading infections (Mardis, 2008). This predicament underscores the urgent need for innovative diagnostic techniques that can adapt to the rapid evolution of antibiotic resistance.

The integration of techniques like MALDI-TOF MS with sophisticated data analysis algorithms has been shown to expedite the identification of resistant strains (Garcia et al., 2024). A retrospective clinical case study involving 63 patients revealed that adopting such methodologies would have altered the clinical management of nine patients, benefiting eight of them (89%). Consequently, machine learning based on MALDI-TOF mass spectrometry emerges as an essential new tool for therapy optimization and antibiotic stewardship (Theodosiou and Read, 2023). Deep learning algorithms, such as Convolutional Neural Networks (CNN) and Long Short-Term Memory (LSTM) networks, can perform rapid and accurate antibiotic susceptibility testing by classifying bacteria into active or non-active strains (Weis et al., 2022).

AI technologies, encompassing machine learning and natural language processing, enable the processing of vast quantities of genomic data, which leads to the identification of resistance-associated genetic mutations and evolutionary patterns (Yu et al., 2018). This capability not only enhances our understanding of how bacteria develop drug resistance but also provides invaluable insights for novel drug development (Zhou and Troyanskaya, 2015). Furthermore, machine learning models have the potential to predict mutational trends and resistance to specific drugs, thereby aiding clinicians in selecting the most effective treatment regimens (Gupta et al., 2021).

#### 4.1.3 Application of artificial intelligence in pathogen transmission and control

Artificial intelligence demonstrates significant potential in monitoring and controlling pathogen transmission. By employing machine learning to recognize transmission patterns, it provides vital decision support for public health authorities, enabling the implementation of more effective outbreak control strategies (Májek et al., 2021). AI can analyze historical epidemic data to predict future disease transmission (Ren et al., 2023). Additionally, AI technologies are utilized for real-time monitoring of infection trends, allowing rapid responses to outbreaks. AI-driven warning systems enhance the predictive capacity for future outbreaks, improving resource allocation and management strategies (Vahedi et al., 2021). These technologies provide scientific evidence for disease control and prevention, bolstering the resilience of public health systems.

#### 4.1.4 Application of artificial intelligence in pathogen genomics

Deep learning, as a crucial AI technology, offers new perspectives and tools for analyzing diversity and evolution in pathogen genomics research. Xu et al. employed deep learning algorithms to efficiently identify various antimicrobial peptides from metagenomic data, significantly advancing the development of next-generation antimicrobials (Wang et al., 2024). AI’s ability to analyze large volumes of genomic sequence data allows it to identify and compare genetic characteristics of diverse microorganisms, revealing their evolutionary relationships and functional traits (Xu et al., 2020). This robust potential for genome annotation and functional prediction provides vital support for microbial ecology and functional research (Angly et al., 2006). Furthermore, constructed databases allow for in-depth exploration of complex interactions between microorganisms and environmental contexts (Sun et al., 2023). This provides valuable insights for research in microbial genomics, ecological analysis, and disease prevention.

#### 4.1.5 Application of artificial intelligence in optimization of treatment strategies

Artificial intelligence is playing an increasingly important role in optimizing treatment strategies for pathogens. By analyzing clinical data, AI can predict the efficacy of various treatment regimens and adjust strategies in real-time according to individual patient condition changes. Li Jinquan utilized AI to identify differences in high-dimensional features of antimicrobial candidate proteins, discovering the best-in-class lytic enzyme LLysSA9, effective in treating bovine mastitis and combating *Staphylococcus aureus* infections (Hirose et al., 2024). This personalized medicine approach not only enhances treatment outcomes but also reduces unnecessary treatments and potential side effects. AI-powered decision support systems integrate medical literature, patient data, and clinical trial results to provide scientific foundations for optimizing treatment plans (Wong et al., 2023).

Moreover, AI excels in drug repurposing and new drug development, using models to simulate the effects of different drugs on pathogens, thereby advancing personalized treatment (Zhang et al., 2024). Liu G et al. highlighted challenges in discovering new antibiotics against *Acinetobacter baumannii* through traditional screening methods, while James J. Collins and colleagues utilized machine learning to screen approximately 7,500 molecules, swiftly identifying those inhibiting *A. baumannii* growth *in vitro* (Melo et al., 2021). Khaledi et al. predicted antimicrobial susceptibility based on genomic and transcriptomic markers, enhancing diagnostic performance by identifying resistance characteristics early in disease progression (Liu et al., 2023).

#### 4.1.6 Application of artificial intelligence in ecology studies of pathogens

The application of artificial intelligence in ecological studies of pathogens opens up new avenues for understanding the ecological roles of microorganisms in various environments (Khaledi et al., 2020). Neural network technology, in particular, demonstrates remarkable performance in this domain. For instance, the vedoNet neural network algorithm, developed by Ananthakrishnan et al., integrates microbiome and clinical data and achieves superior classification capability for clinical remission in inflammatory bowel disease (IBD). Detailed research indicates that early trajectories of microbiome changes can serve as markers for treatment response (Lopatkin and Collins, 2020). Additionally, machine learning and data mining techniques are extensively applied to model and predict microbial community behavior under various environmental conditions, thus helping to reduce disease incidence associated with environmental changes (Ananthakrishnan et al., 2017). This interdisciplinary research not only enhances the understanding of microbial ecology but also provides a scientific foundation for formulating effective environmental management strategies.

#### 4.1.7 Application of artificial intelligence in vaccine development

Traditional vaccine development has largely relied on laborious experimental methods that, while effective, are often time-consuming and have limited success rates (Ai et al., 2020). Recently, data mining and big data analytics have paved new pathways for vaccine development, with artificial intelligence (AI) revolutionizing the field as a tool for antigen selection and immunogen design (Brisse et al., 2020). By utilizing advanced algorithms, AI extracts crucial data from extensive genomic datasets, protein structure information, and immune system interactions, quickly identifying potential vaccine candidate antigens (Aswathy and Sumathi, 2024). For example, AI-driven neural network prediction models trained on a large dataset of over 24,000 peptides can accurately recognize key epitopes detected by the immune system. Prioritizing these epitopes and recommending experimental validation allows AI to significantly shorten the discovery time while minimizing resource investment (Olawade et al., 2024).

By integrating AI algorithms with experimental validation and clinical trials, the vaccine development process is substantially accelerated. This data-driven approach enhances vaccine development efficiency and demonstrates significant potential during global health crises (Ward et al., 2021). During the COVID-19 pandemic, AI played a crucial role in quickly identifying novel antigens through detailed data mining, providing essential support for the rapid development of vaccines (Brisse et al., 2020). In mRNA-based COVID-19 vaccines, AI not only optimized vaccine sequences but also effectively screened delivery vectors, improving overall research and development efficiency (Federico et al., 2023).

#### 4.1.8 Application of artificial intelligence in data analysis and management of pathogens

With the explosion of data volume, the application of AI in image data processing technology for pathogen detection becomes increasingly critical (Zhang et al., 2023). Traditional detection methods, such as nucleic acid and immunological assays, are often time-consuming and complex (Haymond and McCudden, 2021). Through the incorporation of machine learning, particularly deep convolutional neural network (CNN)-based image processing algorithms, AI can rapidly process and analyze microscopic image data, automatically identifying pathogens, thus significantly reducing diagnostic time. For instance, Rahman et al. utilized the DenseNet CNN model to classify 89 fungal genera from microscopic images, achieving a prediction accuracy of 65.35% (Whiley and Taylor, 2016), marking a notable enhancement in detection efficiency. Tao Chenglong integrated the HMI system with Buffer Net, developing a CNN-based AI-assisted system for rapid and automatic bacterial identification (Rahman et al., 2023). Additionally, Devan et al. employed a transfer learning method based on CNN, requiring minimal preprocessing to detect HCMV nucleocapsids in TEM images (Tao et al., 2022). In tuberculosis detection, Kuok et al. attained an 86% detection rate using a region-refined Faster R-CNN algorithm to automatically detect acid-fast bacilli on slides, significantly outperforming the traditional support vector machine (SVM) method, which had a detection rate of 70.93% (Shaga Devan et al., 2021). Chung et al. combined MALDI-TOF MS (matrix-assisted laser desorption ionization-time of flight mass spectrometry) with CNN technology for the rapid identification of hemolytic streptococci, quickly pinpointing infection sources, effectively preventing epidemic spread, and providing robust technical support for public health management (Kuok et al., 2019).

### 4.2 Interconnections among topics

As illustrated in Figure 7, there is significant lexical overlap among the various research topics, reflecting a strong interconnection and a trend towards interdisciplinary integration in the field. In the topic modeling analysis, drug resistance (Topic 1) and vaccine development (Topic 6) exhibited the highest weights (0.226 and 0.195, respectively). The growing global challenge of bacterial drug resistance and the threat from emerging infectious diseases in recent years have heightened the need for large-scale immunization efforts. AI contributes to the rapid development of vaccines by accelerating antigen identification and predicting immune responses.

Genomics research (Topic 3) and drug resistance research (Topic 1) are closely linked through shared genetic analysis methods. Genomics plays a critical role in drug resistance research; AI can swiftly analyze genomic sequencing data to identify and classify antibiotic resistance genes (Qu et al., 2019), and this genomic data can be integrated into machine learning models to predict antibiotic sensitivity and resistance phenotypes (Chung et al., 2019).

The word cloud also reveals a synergy between transmission control (Topic 2) and ecological research (Topic 5), particularly in environmental monitoring. For example, combining AI algorithms to develop predictive models can forecast high- and low-risk areas for pathogen outbreaks under future climate conditions. This approach is especially effective when linking climatology research (analyzing factors such as temperature and precipitation) with ecological studies (focusing on pathogen vectors or hosts), thereby significantly enhancing the predictive accuracy and interpretability of these models, enabling precise control and prevention (Melo et al., 2021; Farooq et al., 2022).

Data analysis and management (Topic 7) appears to be a crucial link across all research topics. Data analysis and management is not merely an independent theme but rather a key element throughout the pathogen research process. AI algorithms heavily depend on the quality of pathogen data and metadata to enhance research accuracy and reliability. From pathogen detection to predicting antibiotic resistance and optimizing treatments, substantial amounts of genomic sequencing data, electronic health records, and other clinical data are collected, processed, and analyzed, forming the training datasets for machine learning models.

### 4.3 Practical applications

The application of artificial intelligence in pathogen research is gradually transitioning from laboratory research to clinical practice, with official approval in certain regions. For example, the U.S. Food and Drug Administration (FDA) has approved Clever Culture Systems’ APAS Compact system for the automated assessment of plates in clinical microbiology laboratories, demonstrating high sensitivity and specificity in detecting urine cultures (Peiffer-Smadja et al., 2020).

Many hospitals have already implemented AI for pathogen detection. Taiwan’s Tri-Service General Hospital, along with four secondary hospitals, has successfully deployed a solution powered by an AI clinical decision support system (AI-CDSS) to expedite the detection of carbapenem-resistant *Klebsiella pneumoniae* (KP). This system integrates MALDI-TOF MS technology with machine learning algorithms, accelerating the prediction of bacterial resistance—particularly to carbapenems and colistin—by 1 day compared to traditional antibiotic susceptibility tests (AST). It provides healthcare professionals with resistance probability scores through a web interface, enabling rapid and informed treatment decisions (Ali et al., 2024). Massachusetts General Hospital employs AI to assess the risk of *Clostridium difficile* infections. In a multicenter study involving at least nine hospitals, Dascena’s machine learning algorithms have been used for early sepsis detection and stratification, antimicrobial prescription recommendations, and resistant microorganism colonization predictions, demonstrating the potential to reduce hospital mortality rates, shorten hospital stays, and decrease 30-day readmission rates (Jian et al., 2024; Shimabukuro et al., 2017; Burdick et al., 2020).

AI and machine learning (ML) technologies are also extensively applied in addressing healthcare-associated infections (HAIs). AI systems are capable of predicting surgical site infections (SSIs), hospital-acquired pneumonia (HCAP), and hospital-acquired urinary tract infections (HA-UTI) (McCoy and Das, 2017). For instance, a machine learning model monitoring SSI in colon surgeries has reduced manual workload by 83.9% (Radaelli et al., 2024). A new AI-based training and monitoring system (AITMS) has improved personal protective equipment (PPE) wearing and doffing skills, successfully reducing pathogen infection rates from 1.31 to 0.58% in a Japanese hospital (Cho et al., 2024). The University of Iowa Hospitals and Clinics utilized machine learning to decrease surgical site infection rates by 74%, while Philips’ “Connected Care” system reduced detection time for nosocomial infections by 87% (Huang et al., 2023).

Artificial intelligence has also played a practical role in global public health. Systems like HealthMap utilize natural language processing to analyze online news and professional resources, providing global alert information for outbreaks such as the Middle East respiratory syndrome coronavirus (MERS-CoV) and severe acute respiratory syndrome coronavirus 2 (SARS-CoV-2) (Agrebi and Larbi, 2020; Ali et al., 2023). The U.S. CDC employs machine learning models to predict influenza trends (Hossain and Househ, 2016). During the COVID-19 pandemic, AI technologies were implemented in genomic classification, lineage mapping, and optimization of testing strategies. The ZOE COVID Study collected symptom data via a smartphone app, offering invaluable insights for public health (Reich et al., 2019). Singapore airport implemented thermal imaging for temperature monitoring of potential infections, combining physiological parameters with advanced analytical methods to classify high-risk influenza patients (Menni et al., 2020).

### 4.4 Opportunities and challenges

The real-world application of artificial intelligence (AI) in pathogen research is still in its infancy; however, it reveals immense potential for development while facing numerous challenges and obstacles. The following are four key directions for enhancing AI application in this field:

Advanced Machine Learning Algorithms: With the increase in computational power and data accumulation, more sophisticated and accurate deep learning models can be applied to pathogen research to improve the accuracy of disease prediction and enhance the capability to handle multidimensional data.
Richer Sample Data: By collecting additional sample data from diverse clinical settings worldwide, AI systems can improve their generalization ability, thereby increasing their robustness across varied medical environments.
User-Friendly Interface Design: Developing intuitive and easy-to-use interfaces, along with providing adequate training for healthcare professionals, can significantly promote the widespread application of AI technologies in clinical practice.
Application of Extreme Value Theory: Integrating extreme value theory with robust statistical methods in epidemiology and public health can aid in the early detection of anomalies in transmission dynamics. This is particularly beneficial for the early warning of rare infectious events, such as emerging infectious diseases, providing strong support for public health interventions.

However, several challenges must be overcome to advance AI applications:

High Costs: The development, deployment, and maintenance of AI models are capital-intensive. Solutions include utilizing open-source AI tools and models and creating government subsidy policies to lower the barrier to technology access.
Training and Talent Shortage: Healthcare professionals require appropriate training to effectively use AI tools. This issue can be addressed by implementing targeted AI training programs and cultivating more medical professionals with expertise in AI.
Data Quality and Accessibility: High-quality data is crucial for training AI models. Challenges can be tackled by establishing standardized data-sharing mechanisms, improving data collection and annotation methods, and enhancing data security and privacy protections.
Ethical and Legal Issues: The use of AI in medical decision-making involves ethical and legal responsibilities. This necessitates the development of ethical guidelines and legal regulations for AI applications, clearly defining accountability and establishing effective oversight mechanisms to ensure lawful and compliant use of AI systems.
Model Explainability: The “black box” nature of AI models affects their applicability and acceptance in clinical practice. Therefore, developing more interpretable AI models can help clinicians understand their decision processes, thereby increasing trust and encouraging their use (Sun et al., 2015; Hassija et al., 2024).

## 5 Conclusion

In this study, we conducted a comprehensive analysis of the application of artificial intelligence (AI) in pathogenic microbiology research using bibliometrics and topic modeling. We examined 27,420 relevant publications from 2010 to 2024, uncovering an exponential growth trend in publications since 2016, primarily focused on eight key areas: pathogen detection, antibiotic resistance prediction, transmission and control, genomic analysis, therapeutic optimization, ecological studies, vaccine development, and data management systems.

The results from topic modeling indicate that the application of AI in pathogen research has become diverse and specialized. For instance, in pathogen detection, AI has significantly improved diagnostic speed and accuracy through the integration of multimodal data fusion technologies. In the realm of antibiotic resistance prediction, machine learning and deep learning models have expedited the identification and analysis of resistance genes. In vaccine development, AI has facilitated rapid progress in antigen recognition and immunogen design, thus playing a critical supportive role in the development of COVID-19 vaccines.

Despite AI’s substantial potential in pathogenic microbiology research, its practical implementation remains in the early stages and faces numerous challenges. Key factors limiting effective AI application include the acquisition and sharing of high-quality data, AI system interpretability, ethical and legal responsibilities, and the high cost of development. To foster further advancements in this field, we recommend strengthening interdisciplinary collaboration to enrich AI model training data, enhancing the user-friendliness of AI tools to promote their adoption and application in clinical practice, and supporting policies to reduce the economic barriers to AI utilization. Addressing these issues collaboratively will enable a fuller realization of AI technologies in tackling challenges in the field of pathogenic microbiology, ultimately contributing to the resilience of health management and public health systems and providing unprecedented opportunities to address global public health challenges.

## Data Availability

The original contributions presented in the study are included in the article/supplementary material, further inquiries can be directed to the corresponding authors.
